# Bosutinib‐associated interstitial lung disease and pleural effusion: A case report and literature review

**DOI:** 10.1002/ccr3.3164

**Published:** 2021-05-07

**Authors:** Qiuying (Selina) Liu, Nour Ali Ass'ad, Cecilia Arana Yi

**Affiliations:** ^1^ University of New Mexico School of Medicine Albuquerque NM USA; ^2^ University of New Mexico Comprehensive Cancer Center Albuquerque NM USA

**Keywords:** bosutinib, bosutinib‐related interstitial lung disease, pleural effusion, tyrosine kinase inhibitors

## Abstract

Bosutinib is a tyrosine kinase inhibitor approved for the management of chronic myeloid leukemia (CML). Interstitial lung disease and pleural effusion are pulmonary side effects of TKIs rarely associated with bosutinib treatment.

## Graphical abstract

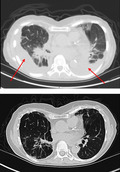

## 1 INTRODUCTION

Bosutinib is a tyrosine kinase inhibitor (TKI) approved for the management of chronic myeloid leukemia (CML). TKIs are associated with pulmonary complications but are rarely described with bosutinib. Here, we report the first description of bosutinib‐induced interstitial lung disease and pleural effusion, which resolved after the discontinuation of bosutinib.

Tyrosine kinase inhibitors targeting BCR/ABL have revolutionized the treatment of CML, changing its prognosis from an overall survival of 5 years to near normal life expectancy.^1^, ^2^ However, TKIs are associated with potentially serious complications, such as pleural effusion, pneumonitis, and vascular and metabolic disorders.^3^, ^4^ Bosutinib is a BCR/ABL inhibitor used in frontline treatment of CML or in treatment of chronic, accelerated, or blast phase CML resistant or intolerant to prior TKIs.^1^, ^2^, ^5^, ^6^ The most common side effects are gastrointestinal, followed by thrombocytopenia and abnormal liver function tests.^6^ However, there have been cases reporting pulmonary injury related to bosutinib.^7^ Here, we discuss a case of bosutinib‐induced interstitial lung disease and pleural effusion.

## 2 CASE HISTORY/EXAMINATION

A 71‐year‐old woman with no history of significant comorbidities develops the diagnosis of chronic phase CML in 2000. She received frontline treatment with imatinib and achieved major molecular remission for 14 years. Her treatment course was interrupted for 2 months in 2007 by pancreatitis and for 1 month in 2008 after she developed nitrofurantoin‐induced nonspecific interstitial lung disease. With the use of nocturnal oxygen at 2 L/min on nasal cannula, discontinuation of the nitrofurantoin, and 1 month of prednisone, her respiratory condition almost resolved. In May of 2014, the patient's disease progressed to hematological and cytogenetic relapse, for which she started treatment with a 2‐month course of dasatinib, followed by a 4‐month course of nilotinib. Both treatments required dose reductions and interruptions leading to discontinuation because of grade 3 gastrointestinal and dermatological side effects. The patient did ultimately achieve early molecular response after nilotinib treatment. No BCR/ABL1 kinase domain mutation analysis was performed at the time of nilotinib discontinuation. While on early molecular response, in January of 2015, the patient started bosutinib at a daily dose of 500 mg orally. Within 1 month, the patient developed symptoms of diarrhea, nausea, and vomiting that resolved after bosutinib dose reduction to 400 mg. She achieved complete hematological, cytogenetic remission, and a major molecular response (MMR) (BCR‐ABL1 < 0.1%) within the first year. She continued with MMR until the development of respiratory complications. After 55 months of bosutinib and without any changes in her respiratory status since 2014, the patient reported in August of 2019 progressive dyspnea and dry cough, requiring an increase in her oxygen requirements from 2 to 4 L/min through a nasal cannula.

**FIGURE 1:**
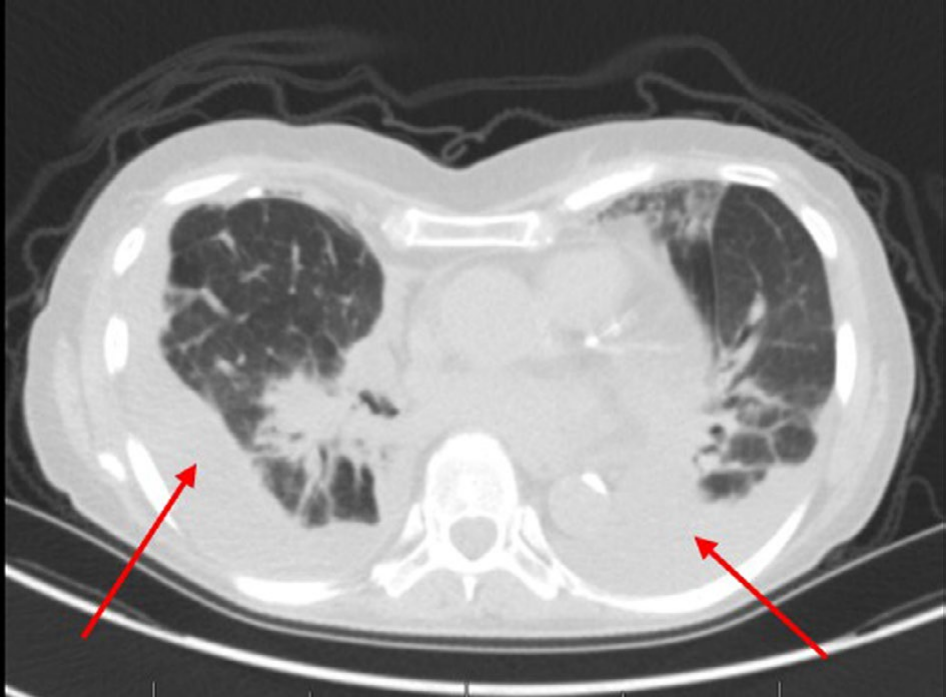
New bilateral pleural effusions, (red arrows) peribronchovascular ground‐glass opacities, and mild bilateral centrilobular tree‐in‐bud nodules

**FIGURE 2:**
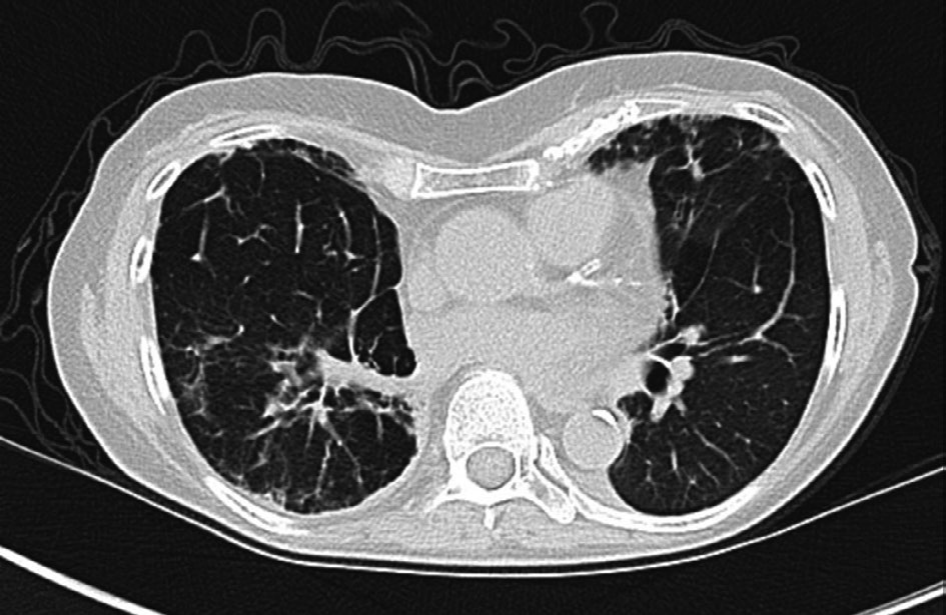
Resolution of pleural effusion and decreased peribronchovascular ground‐glass opacities

Aside from taking pantoprazole, she denied any environmental, occupational, or other drug exposures. The pulmonary function test revealed restrictive ventilatory defects and decreased diffusing capacity (Table 1). The echocardiogram was normal. Computed tomography (CT) of the chest showed nonspecific chronic interstitial lung disease and new moderate bilateral pleural effusions with a small pericardial effusion (Figure 1). An autoimmune panel was negative. The bronchoalveolar lavage was negative for infection with a bland count. The right diagnostic thoracentesis showed sterile exudate with 88% lymphocytes concerning for a drug reaction. Pleural fluid cultures and cytologies were negative. The bronchoscopy and CT scan findings raised the suspicion of bosutinib‐induced interstitial lung disease and pleural effusion. In October of 2019, after bronchoscopy results, the pulmonary specialist recommended the discontinuation of bosutinib without starting steroids. In January of 2020, the patient noted significant improvement in her cough and dyspnea and was no longer requiring oxygen treatment. The chest CT scan in January 2020 showed almost complete resolution of pleural and pericardial effusions and decreased peribronchovascular ground‐glass opacities (Figure 2). Repeat spirometry showed improved pulmonary function (Table 1) in January of 2020. At the same time of symptom improvement, the patient presented with hematological and molecular relapse. In March of 2020, she initiated treatment with ponatinib. In May of 2020, the patient reported symptomatic uncontrolled hypertension requiring the temporary holding of the drug.

**Table 1:** Pulmonary function tests

	4/2019	% predicted	10/2019	% predicted	1/2020	% predicted
Forced vital capacity (FVC)	1.12 L	47%	0.83 L	35%	1.03	44%
Forced expiratory volume (FEV1)	0.97 L	54%	0.81 L	45%	1	56%
FEV1:FVC	–	86%	–	98%	–	97%
Diffusing capacity	6 L	31%	4 L	21%	9.25 L	47%

## 3 DISCUSSION

The prognosis of CML has changed from a fatal hematological malignancy to a curable disease thanks to the use of BCR‐ABL TKIs. Since most patients will require treatment indefinitely, it is essential to understand the potential risks of such medications. TKI selectivity is often a critical issue as most TKIs have inhibitory actions against a wide variety of kinases.^8^ Since multiple signaling pathways contribute to tumor pathogenesis, inhibiting several kinases simultaneously sometimes represents an advantage. The overall toxicity of TKIs correlates with the nonselective inhibition of several kinases.^9^ Risk factors for the adverse effects include a higher dose, a longer duration of treatment, older age, and a history of cardiac disease.^10^

Among the different BCR‐ABL TKIs, dasatinib is the drug most related to pulmonary toxicities.^11^ Dasatinib‐induced pleural effusion has an incidence of 15%‐35%.^4^, ^12^, ^13^ Other pulmonary complications, such as pulmonary arterial hypertension (PAH) and interstitial pneumonitis, have also been reported.^2^, ^10^, ^12^, ^14^

Bosutinib is uncommonly associated with pulmonary side effects. In a long‐term safety study on bosutinib, the incidence of pleural effusions ranged between 4% and 8%, with a median time to onset of 33 months after initiation of treatment.^10^, ^13^, ^14^, ^15^ Tiribelli reported a 30% incidence of pleural effusion after similar complications post dasatinib^16^; moreover, the incidence raises up to 52% in patients using bosutinib as forth‐line therapy of CML with previous pleural effusion after dasatinib.^17^ Moguillansky et al reported a case of bosutinib‐associated pleural effusions that resolved after discontinuation of the medication.^7^ Jutant et al reported a case of bosutinib‐related pneumonitis.^12^ As in the case we are presenting, bosutinib may also be associated with interstitial lung disease and pleural effusion; a spectrum of lung complications previously described with other TKIs.^18^

Although the mechanism of bosutinib‐induced lung injury is unclear, it could be causing oxidative and reticular endothelium stress, similar to dasatinib. Guignabert et al's studies showed that dasatinib mediated endothelial cell dysfunction and vascular damage in a dose‐dependent manner.^19^ The risk factors for bosutinib‐induced lung complications are a higher dosage, longer therapy duration, older age, history of cardiac disease, hypertension, or hypercholesterolemia.^4^, ^9^, ^10^ Patients on TKIs need careful monitoring of respiratory symptoms such as rapid weight gain, progressive dyspnea, or nonproductive cough. A physical examination result suggestive of pleural effusion includes dull percussion or focally diminished breath sounds. This requires prompt confirmation with imaging studies. Depending on the size of the effusion and the index of suspicion for possible pleural space infection, a thoracentesis confirms the diagnosis. Typically, pleural fluid in drug‐induced pleural effusion is exudative with lymphocyte predominance,^20^ as in our patient's case. The management of TKI‐associated pleural effusion depends on the radiographic findings and clinical compromise.^4^, ^21^ If symptoms are minimal, no treatment is necessary. For large symptomatic pleural effusions, diuresis and a short steroid course can be used besides TKI dose reduction or discontinuation (temporarily or permanently), as recommended by the National Comprehensive Cancer Network. (NCCN, Chronic Myeloid Leukemia, v.3.2020).^22,22^

Although interstitial lung disease (ILD) was a nonreported side effect of BCR‐ABL TKI during the pre‐approval clinical trials, case reports in the postmarketing period suggest ILD could be a side effect of BCR‐ABL TKIs.^3^ Peerzada et al showed patients with BCR‐ABL TKIs‐induced ILD often presented with dyspnea, cough, hypoxia, and bilateral ground‐glass opacities.^23^ ILD due to TKIs is a diagnosis of exclusion. A detailed history and physical examination are necessary, and CT chest and sometimes lung biopsy can guide diagnosis. The mechanism of TKIs‐induced pulmonary injury seems to be dose‐dependent and typically reversible with discontinuation of TKI therapy. In some cases, the addition of corticosteroids confers some clinical benefit to avoid TKI therapy cessation.^1^, ^11^ In patients who develop moderate to severe TKI‐associated interstitial lung disease, re‐challenging with alternative TKI therapy requires close monitoring since the risk of recurrence is high.^11^ ^.^

## CONFLICT OF INTEREST

None declared.

## AUTHOR CONTRIBUTIONS

QL: completed the background research and drafted and edited the manuscript. NA: edited the manuscript. CAY: edited the manuscript and approved the final manuscript.

## ETHICAL APPROVAL

The patient provided written informed consent for the publication.
